# Estimating the generation interval for coronavirus disease (COVID-19) based on symptom onset data, March 2020

**DOI:** 10.2807/1560-7917.ES.2020.25.17.2000257

**Published:** 2020-04-30

**Authors:** Tapiwa Ganyani, Cécile Kremer, Dongxuan Chen, Andrea Torneri, Christel Faes, Jacco Wallinga, Niel Hens

**Affiliations:** 1 I-BioStat, Data Science Institute, Hasselt University, Hasselt, Belgium; 2 Centre for Infectious Disease Control, National Institute for Public Health and the Environment, Bilthoven, the Netherlands; 3 Leiden University Medical Center, Leiden, the Netherlands; 4 Centre for Health Economics Research and Modelling Infectious Diseases (CHERMID), Vaccine and Infectious Disease Institute, University of Antwerp, Antwerp, Belgium

**Keywords:** COVID-19, generation interval, serial interval, incubation period, reproduction number

## Abstract

**Background:**

Estimating key infectious disease parameters from the coronavirus disease (COVID-19) outbreak is essential for modelling studies and guiding intervention strategies.

**Aim:**

We estimate the generation interval, serial interval, proportion of pre-symptomatic transmission and effective reproduction number of COVID-19. We illustrate that reproduction numbers calculated based on serial interval estimates can be biased.

**Methods:**

We used outbreak data from clusters in Singapore and Tianjin, China to estimate the generation interval from symptom onset data while acknowledging uncertainty about the incubation period distribution and the underlying transmission network. From those estimates, we obtained the serial interval, proportions of pre-symptomatic transmission and reproduction numbers.

**Results:**

The mean generation interval was 5.20 days (95% credible interval (CrI): 3.78–6.78) for Singapore and 3.95 days (95% CrI: 3.01–4.91) for Tianjin. The proportion of pre-symptomatic transmission was 48% (95% CrI: 32–67) for Singapore and 62% (95% CrI: 50–76) for Tianjin. Reproduction number estimates based on the generation interval distribution were slightly higher than those based on the serial interval distribution. Sensitivity analyses showed that estimating these quantities from outbreak data requires detailed contact tracing information.

**Conclusion:**

High estimates of the proportion of pre-symptomatic transmission imply that case finding and contact tracing need to be supplemented by physical distancing measures in order to control the COVID-19 outbreak. Notably, quarantine and other containment measures were already in place at the time of data collection, which may inflate the proportion of infections from pre-symptomatic individuals.

## Introduction

The 2019 coronavirus disease (COVID-19) outbreak that started in Wuhan, China in December 2019 has now been declared a pandemic. As at 22 April 2020, 2,573,143 cases of COVID-19 have been confirmed in 185 countries and territories around the world [1]. In order to plan intervention strategies aimed at bringing disease outbreaks such as the COVID-19 outbreak under control as well as to monitor disease outbreaks, public health officials depend on insights about key disease transmission parameters that are typically obtained from mathematical or statistical modelling. Examples of key parameters include the reproduction number (*R*) (average number of infections caused by an infectious individual), and distributions of the generation interval (time between infection events in an infector-infectee pair), serial interval (time between symptom onsets in an infector-infectee pair) and incubation period (time between moment of infection and symptom onset) [2]. Estimates of the reproduction number together with the generation interval distribution can provide insight into the speed with which a disease will spread. On the other hand, estimates of the incubation period distribution can help guide determining appropriate quarantine periods.

As soon as line lists were made available, statistical and mathematical modelling was used to quantify these key epidemiological parameters. Li et al. [3] estimated the basic reproduction number using a renewal equation to be 2.2 (95% confidence interval (CI): 1.4–3.9), the serial interval distribution to have a mean of 7.5 days (95% CI: 5.3–19) based on six observations, and the incubation period distribution to have a mean of 5.2 days (95% CI: 4.1–7.0) based on 10 observations. Other studies estimated the incubation period distribution to have a mean of 6.4 days (95% credible interval (CrI): 5.6–7.7) [4], mean of 5.0 days (95% CrI: 4.2–6.0) [5], mean of 5.2 days (range: 1.8–12.4) [6], and a mean of 4.8 days (range: 2–11) [7].

When the incubation period does not change over the course of the epidemic, the expected values of the serial and generation interval distributions are expected to be equal but their variances to be different [8]. It has recently been shown that ignoring the difference between the serial and generation interval can lead to biased estimates of the reproduction number [8]. More specifically, when the serial interval distribution has larger variance than the generation interval distribution, using the serial interval as a proxy for the generation interval will lead to an underestimation of the effective reproduction number, *R*. When *R* is underestimated, this may lead to prevention policies that are insufficient to stop disease spread [8].

The most well-known method to estimate the serial interval distribution from line list data is the likelihood-based estimation method proposed by Wallinga and Teunis [9]. In 2012, Hens et al. [10] proposed using the expectation-maximisation (EM) algorithm to estimate the generation interval distribution from incomplete line list data based on the method by [9] and allowing for auxiliary information to be used in assigning potential infector-infectee pairs. Te Beest et al. [11] used a Markov chain Monte Carlo (MCMC) approach as an alternative to the EM algorithm, to facilitate taking uncertainty related to the dates of symptom onset into account. In this paper, we use a MCMC approach to estimate, next to the serial interval distribution, the generation interval distribution upon specification of the incubation period distribution. We compare the impact of differences among previous estimates of the incubation period distribution for COVID-19.

## Methods

### Data sources

The data used in this paper are symptom onset dates and cluster information for confirmed cases in Singapore (21 January to 26 February 2020) and Tianjin, China (14 January to 27 February 2020).

As at 26 February, 91 confirmed COVID-19 cases had been reported in Singapore. Detailed information on age, sex, known travel history, time of symptom onset and known contacts was available for 54 of these cases from the Ministry of Health (https://www.moh.gov.sg/news-highlights/, last accessed 26 February). For cases with no infector information available, it was assumed that they could have been infected by any other case within the same cluster. There were four clusters in these data, i.e. Grace Assembly of God church, Grand Hyatt business meeting, Seletar Aerospace Heights construction site and Yong Thai Hang shop. Cases known to be Chinese/Wuhan nationals or known to have been in close contact with a Chinese/Wuhan national were labelled as index cases. All other cases were assumed to have been infected locally.

As at 27 February, 135 confirmed cases had been reported by the Tianjin Municipal Health Commission. Data on these cases were available in official daily reports (http://www.tjbd.gov.cn/zjbd/gsgg/, last accessed 27 February) and included age, sex, relationship to other known cases, and travel history to risk areas in and outside Hubei Province, China. In these data, 114 cases can be traced to one of 16 clusters. The largest cluster consisting of 45 cases could be traced to a shopping mall in Baodi district of Tianjin. Through contact investigations, potential transmission links were identified for cases who had close contacts. Travel history information was used to identify some individuals as imported cases. For cases with no infector information available, it was assumed that they could have been infected by any other case within the same cluster.

### Model

For *i =* 2,…,*n*, denote *t_i_* the time of infection for individual *i*, *t_v(i)_* the time of infection for the infector of individual *i*, *δ_i_* the incubation period for individual *i* and *δ_v(i)_* the incubation period for the infector of individual *i*. The serial interval (*Z_i_*) for case *i* is a linear combination of latent variables, i.e. *Z_i_* = (*t_i_* + *δ_i_*) – (*t_v(i)_* + *δ_v(i)_*). Assuming the incubation period is independent of the infection time, *Z_i_* can be rewritten as a convolution of the generation interval for individual *i* and the difference between the incubation period of individual *i* and the incubation period of its infector *v(i)* [8], i.e.,

$$Z_{i}=\left(t_{i}+\delta_{i}\right)-\left(t_{v\left(i\right)}+\delta_{v\left(i\right)}\right)$$

$$=\left(t_{i}-t_{v\left(i\right)}\right)+\left(\delta_{i}-\delta_{v\left(i\right)}\right)$$

$$=X_{i}+Y_{i}$$ (1)

The random variables *X_i_* and *δ_i_* are positive and are both assumed to be independent and identically distributed, i.e. *X_i_ ~ f*(*x*; *Θ*_1_) and *δ_i_ ~ k*(*δ; Θ*_2_), so that *Y_i_ ~ g*(*y_i_*; *Θ*_2_). Formula (1) implies that both the generation interval and serial interval distributions have the same mean and that the latter has a larger variance and can be negative.

The observed serial interval, *z_i_*, can be expressed in terms of the latent variables as *z_i_* = *x_i_* + *y_i_*, which implies that, *z_i_ ~ h*(*z_i_*; *Θ*_1_, *Θ*_2_). The density function *h(.)* is given by Mood et al. [12],

$$h\left(z;\Theta_{1},\Theta_{2}\right)=\int_{-\infty }^{\infty }f\left(z-y;\Theta_{1}\right)g\left(y;\Theta_{2}\right)dy.$$

In general, *h*(*z*;*Θ*_1_, *Θ*_2_) and *g*(*y*; *Θ*_2_) have no closed form for arbitrary choices of *f*(*x*; *Θ*_1_) and *k*(*δ*; *Θ*_2_). Monte Carlo methods [13] can be used to estimate *h*(*z*; *Θ*_1_, *Θ*_2_) as follows,

$$h(z;\Theta_{1},\Theta_{2})=\int_{-\infty }^{\infty }f(z-y;\Theta_{1})g(y;\Theta_{2})dy$$

$$=\;E_{Y}\left[f\left(z-y;\Theta_{1}\right)\right]$$

$$=\;\frac{1}{J}\sum_{j=1}^{J}f(z-y_{j};\Theta_{1}),$$

where *J* is the number of Monte Carlo samples (i.e. 300) and *y_j_* is the *j^th^* Monte Carlo sample drawn from *g*(*y*; *Θ*_2_). When all infector-infectee pairs are observed, the likelihood function is given by,

$$L\left(\Theta |z_{i},\;v\left(i\right)\right)\;=\;\prod_{i=2}^{n}\frac{1}{J}\sum_{j=1}^{J}f\left(z_{i}-y_{j}|\Theta \right),$$

where *Θ* = [*Θ*_1_, *Θ*_2_] [8]. To account for uncertainty in the transmission links we resort to a Bayesian framework in which missing links are imputed [11] (see the following section, ‘Parameter estimation’). The likelihood function is then given by *L* (*Θ*,*v*(*i*)*^missing^* |*z_i_*, *v*(*i*)). In the main analyses missing links *v*(*i*)*^missing^* are imputed allowing for positive serial intervals only. As a sensitivity analysis, we do not impose any constraints on whether or not serial intervals have a positive value.

### Parameter estimation

We use the Bayesian method described in te Beest et al. [11] for parameter estimation. This method proceeds in two steps. The first step updates the missing links *v*(*i*)*^missing^* and the second step updates the parameter vector *Θ*_1_, i.e. the parameters of the generation interval distribution. We assume that both the generation interval and the incubation period are gamma distributed, i.e. *f*(*x*; *Θ*_1_) ≡ *Γ*(*α*_1_, *β*_1_) and *k*(*δ*; *Θ*_2_) ≡ *Γ*(*α*_2_, *β*_2_). The parameter vector *Θ*_2_ is fixed to (*α*_2_ = 3.45; *β*_2_ = 0.66), corresponding to an incubation period with a mean of 5.2 days and a standard deviation (SD) of 2.8 days [6]. Minimally informative uniform priors are assigned to the parameters of the generation interval distribution, i.e. *α*_1_ *~ U*(0,30) and *β*_1_ *~ U*(0,20). For cases with multiple potential infectors, the possible links *v*(*i*)*^missing^* are assigned equal prior probabilities. The missing links are updated using an independence sampler, whereas *Θ*_1_ is updated using a random-walk Metropolis-Hastings algorithm with a uniform proposal distribution [13]. We evaluate the posterior distribution using 3,000,000 iterations of which the first 500,000 are discarded as burn-in. Thinning is applied by taking every 200th iteration. The mean and variance of the generation interval distribution are monitored within the MCMC chain. Posterior point estimates are given by the 50% percentiles of the converged MCMC chain. CrIs are given by the 2.5% and 97.5% percentiles of the converged MCMC chain. The serial interval distribution is obtained by simulating 1,000,000 draws from *h*(*z*; *Θ*_1_, *Θ*_2_). All analyses were performed using R software version 3.6.2 (R Foundation, Vienna, Austria), while datasets and code are available on GitHub (https://github.com/cecilekremer/COVID19). 

### Corollary epidemiological parameters

The Figure shows three possible transmission scenarios. The proportion of pre-symptomatic transmission is calculated as *p = P*(*X_i_* < *δ_v(i)_*), i.e. pre-symptomatic transmission occurs when the generation interval is shorter than the incubation period of the infector. This proportion was obtained by simulating values from the estimated generation interval and incubation period distributions, assuming a mean incubation time of 5.2 days [6].

**Figure:**
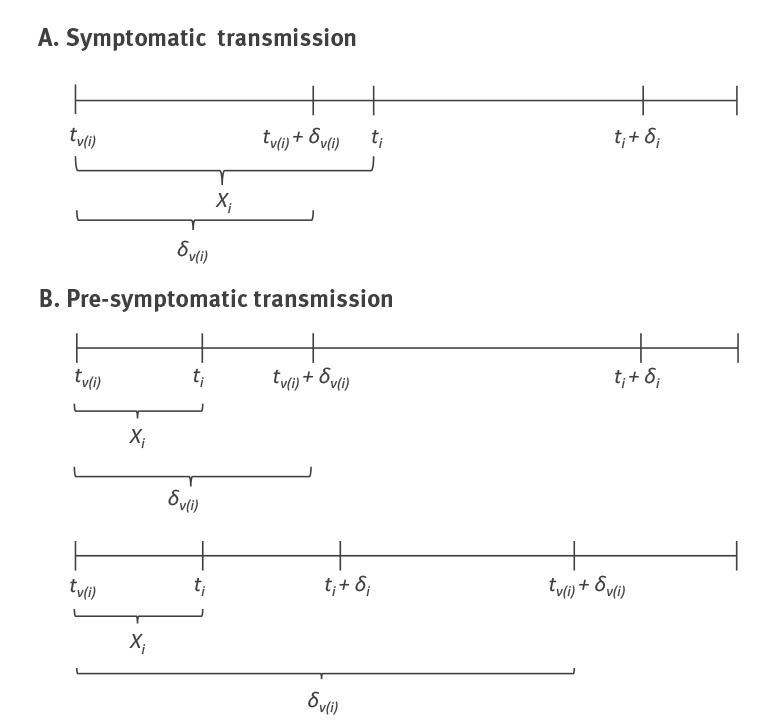
Three possible coronavirus disease (COVID-19) transmission scenarios: (A) one symptomatic transmission scenario and (B) two pre-symptomatic transmission scenarios

The upper figure of panel B shows pre-symptomatic transmission where the infector develops symptoms before the infectee (i.e. positive serial interval), whereas the lower figure shows pre-symptomatic transmission where the infector develops symptoms after the infectee (i.e. negative serial interval). Note that the figure does not include asymptomatic transmission, i.e. infected individuals who may not show symptoms but can transmit infection.

For each of the two outbreaks, i.e. Singapore and Tianjin, *R* is calculated as

$$R=e^{r\mu -\frac{1}{2}r^{2}\sigma^{2}}$$

In this, *r* denotes the exponential growth rate estimated from the early ascending phase of the incidence curve, and *μ* and *σ* ^2^ are the mean and variance of either the generation interval distribution or the serial interval distribution [14]. We calculate *R* in order to highlight the bias that occurs when the serial interval distribution is used as a proxy for the generation interval distribution [8].

CrIs for *p* and *R* are calculated by evaluating *p* and *R* at each iteration of the converged MCMC chain, i.e. at each mean-variance pair of the posterior generation/serial interval distribution. The 95% CrIs are given by the 2.5% and 97.5% percentiles of the resulting distributions.

### Sensitivity analyses

As sensitivity analyses, we investigate the robustness of our estimates of the generation interval distribution to the choice of different incubation period distributions. In particular, we fix *Θ*_2_ to (*α*_2_ = 7.74; *β*_2_ = 1.21) and (*α*_2_ = 4.36; *β*_2_ = 0.91), corresponding to an incubation period with a mean of 6.4 and SD of 2.3 days [4], and a mean of 4.8 and a SD 2.6 days [7], respectively.

In our main, i.e. baseline, analyses, missing serial intervals were only allowed to be positive, i.e. the symptom onset time of the infector has to occur before that of the infectee. However, given that pre-symptomatic transmission is possible, this can be deemed an unrealistic assumption. Therefore, we assess the impact of allowing for negative serial intervals on our estimates of the generation interval distribution.

To further assess the robustness of the estimated generation interval distribution, for each dataset, we fit the model to data from the largest cluster. In the Tianjin dataset, the largest cluster is the shopping mall cluster consisting of 45 cases. In the Singapore dataset, this is the Grace Assembly of God cluster consisting of 25 cases.

## Results

### Estimates of key epidemiological parameters

Table 1 shows parameter estimates of the generation and serial interval distributions for each dataset, assuming an incubation period with a mean of 5.2 days and a SD of 2.8 days. The mean generation time is estimated to be 5.2 days (95% CI: 3.78–6.78) for the Singapore data, and 3.95 days (95% CI: 3.01–4.91) for the Tianjin data. As expected, the estimated means of the generation interval and serial interval distributions are approximately equal, but the latter has a larger variance.

**Table 1:** Parameter estimates and credible intervals of generation and serial interval distributions of COVID-19 using reported information on infector-infectee pairs and assuming an incubation period with a mean of 5.2 and a SD of 2.8 days, Singapore, 21 January–26 February 2020; Tianjin, China, 14 January–27 February 2020

Dataset	Scenario	Interval	Estimate (95% credible interval) (days)	
			Mean	SD
**Singapore^a^**	Baseline	GI	5.20 (3.78 - 6.78)	1.72 (0.91 - 3.93)
		SI	5.21 (−3.35 - 13.94)	4.32 (4.06 - 5.58)
**Tianjin (China)^b^**	Baseline	GI	3.95 (3.01 - 4.91)	1.51 (0.74 - 2.97)
		SI	3.95 (−4.47 - 12.51)	4.24 (4.03 - 4.95)

COVID-19: coronavirus disease; GI: generation interval; SD: standard deviation; SI: serial interval.

^a^ Source: Ministry of Health (https://www.moh.gov.sg/news-highlights/, as at 26 February).

^b^ Source: Tianjin Municipal Health Commission (http://www.tjbd.gov.cn/zjbd/gsgg/, as at 27 February).

### Sensitivity analyses

Table 2 shows parameter estimates of the generation and serial interval distributions for each dataset, assuming incubation periods with a mean of 6.4 and a SD of 2.3 days, or a mean of 4.8 and SD of 2.6 days. The parameter estimates are fairly robust to the specified incubation period distribution, with mean generation times of about 5 days for Singapore and 4 days for Tianjin.

**Table 2:** Parameter estimates and credible intervals of generation and serial interval distributions of COVID-19 with missing serial intervals only allowed to be positive by different incubation periods, Singapore, 21 January–26 February 2020; Tianjin, China, 14 January–27 February 2020

Dataset	Assumed incubation period (days)	Interval	Estimate (95% credible interval) (days)	
			Mean	SD
**Singapore^a^**	Mean 6.4, SD 2.3	GI	5.29 (3.89 - 6.77)	2.08 (0.97 - 4.07)
		SI	5.29 (−2.13 - 13.16)	3.86 (3.40 - 5.21)
	Mean 4.8, SD 2.6	GI	5.19 (3.82 - 6.74)	1.77 (0.91 - 4.11)
		SI	5.19 (−2.86 - 13.45)	4.08 (3.79 - 5.51)
**Tianjin (China)^b^**	Mean 6.4, SD 2.3	GI	4.02 (3.11 - 5.00)	2.29 (1.02 - 3.80)
		SI	4.02 (−4.83 - 13.45)	3.98 (3.41 - 5.00)
	Mean 4.8, SD 2.6	GI	3.95 (3.05 - 4.93)	1.75 (0.77 - 3.35)
		SI	3.95 (−4.60 - 12.73)	4.07 (3.76 - 4.97)

COVID-19: coronavirus disease; GI: generation interval; SD: standard deviation; SI: serial interval.

^a^ Source: Ministry of Health (https://www.moh.gov.sg/news-highlights/, as at 26 February).

^b^ Source: Tianjin Municipal Health Commission (http://www.tjbd.gov.cn/zjbd/gsgg/, as at 27 February).

Table 3 shows parameter estimates of the generation and serial interval distributions obtained when allowing for negative serial intervals in case there is no known infector. Compared with baseline analyses (Table 1), estimates of the mean generation time are smaller when allowing for negative serial intervals. The mean generation time is 3.86 days for Singapore and 2.90 days for Tianjin.

**Table 3:** Parameter estimates and credible intervals of generation and serial interval distributions of COVID-19 when allowing serial intervals to be negative and assuming an incubation period with a mean of 5.2 and a SD of 2.8 days, Singapore, 21 January–26 February 2020; Tianjin, China, 14 January–27 February 2020

Dataset	Scenario	Interval	Estimate (95% credible interval) (days)	
			Mean	SD
**Singapore^a^**	Allowing for all possible negative SI	GI	3.86 (2.22 - 5.60)	2.65 (0.87 - 5.43)
		SI	3.86 (−5.15 - 13.88)	4.76 (4.05 - 6.72)
**Tianjin (China)^b^**	Allowing for all possible negative SI	GI	2.90 (1.85 - 4.12)	2.86 (1.37 - 5.04)
		SI	2.90 (−6.12 - 13.47)	4.88 (4.19 - 6.41)

COVID-19: coronavirus disease; GI: generation interval; SD: standard deviation; SI: serial interval.

^a^ Source: Ministry of Health (https://www.moh.gov.sg/news-highlights/, as at 26 February).

^b^ Source: Tianjin Municipal Health Commission (http://www.tjbd.gov.cn/zjbd/gsgg/, as at 27 February).

Table 4 shows parameter estimates obtained when we fit the model to data from the largest cluster (n = 45). We only show results for the Tianjin dataset because for the Singapore data, there were too few cases (n = 25) and the MCMC chain did not converge. When allowing only positive serial intervals for cases with no known infector, the mean generation time is estimated to be 3.50 days. On the other hand, when allowing for negative serial intervals, it is estimated to be 2.57 days.

**Table 4:** Parameter estimates and credible intervals of generation and serial interval distributions of COVID-19 for the largest cluster under different scenarios for the serial interval and assuming an incubation period with a mean of 5.2 and a SD of 2.8 days, Tianjin, China, 14 January–27 February 2020

Dataset	Scenario	Interval	Estimate (95% credible interval) (days)	
			Mean	SD
**Tianjin (China)** ^a^	Baseline^b^	GI	3.50 (2.10 - 5.03)	1.70 (0.65 - 4.10)
		SI	3.50 (−5.02 - 12.25)	4.31 (4.01 - 5.70)
	Allowing for all possible negative SI	GI	2.57 (1.14 - 4.30)	2.58 (0.68 - 6.11)
		SI	2.57 (−6.28 - 12.70)	4.72 (4.02 - 7.28)

COVID-19: coronavirus disease; GI: generation interval; SD: standard deviation; SI: serial interval.

^a^ Source: Tianjin Municipal Health Commission (http://www.tjbd.gov.cn/zjbd/gsgg/, as at 27 February).

^b^ Baseline is the scenario in which missing serial intervals are only allowed to be positive.

### Estimates of corollary epidemiological parameters

Table 5 shows the proportions of pre-symptomatic transmission and reproduction numbers for each dataset. Pre-symptomatic transmission is higher when allowing for negative serial intervals for cases with no known infector. The reproduction number is lower when estimated using the serial interval compared with when using the generation interval.

**Table 5:** Proportion of pre-symptomatic transmission (*p*) and reproduction number (*R*) of COVID-19 estimated using generation interval or serial interval and assuming an incubation period with a mean of 5.2 and a SD of 2.8 days, Singapore, 21 January–26 February 2020; Tianjin, China, 14 January–27 February 2020

Dataset	Scenario	Interval	Estimate (95% credible interval)	
			*p*	*R*
**Singapore^a^**	Baseline^b^	GI	0.48 (0.32–0.67)	1.27 (1.19–1.36)
		SI	NA^c^	1.25 (1.17–1.34)
	Allowing for all possible negative SI	GI	0.66 (0.45–0.84)	1.19 (1.10–1.28)
		SI	NA^c^	1.17 (1.08–1.26)
**Tianjin (China)^d^**	Baseline	GI	0.62 (0.50–0.76)	1.59 (1.42–1.78)
		SI	NA^c^	1.41 (1.26–1.58)
	Allowing for all possible negative SI	GI	0.77 (0.65–0.87)	1.32 (1.18–1.51)
		SI	NA^c^	1.17 (1.05–1.34)

COVID-19: coronavirus disease; GI: generation interval; NA: not applicable; SI: serial interval.

^a^ Source: Ministry of Health (https://www.moh.gov.sg/news-highlights/, as at 26 February).

^b^ Baseline is the scenario in which missing serial intervals are only allowed to be positive.

^c^ Not applicable as the generation interval estimate is used for calculation the proportion of pre-symptomatic transmission.

^d^ Source: Tianjin Municipal Health Commission (http://www.tjbd.gov.cn/zjbd/gsgg/, as at 27 February).

## Discussion

We estimated the generation time to have a mean of 5.20 days (95% CrI: 3.78–6.78) and a SD of 1.72 days (95% CrI: 0.91–3.93) for the Singapore data, and a mean of 3.95 days (95% CrI: 3.01–4.91) with a SD of 1.51 days (95% CrI: 0.74–2.97) for the Tianjin data. These mean estimates increased only slightly when increasing the mean incubation period. For the Singapore data, allowing the serial interval to be negative decreased the estimated mean generation time from 5.20 days, when restricting missing serial intervals to be positive, to 3.86 days (95% CrI: 2.22–5.60), when allowing them to be negative. For the Tianjin data, the baseline estimate of the mean generation time (3.95 days) is about the same as when allowing serial intervals to be negative in the Singapore data. However, there were already some negative serial intervals among the reported links in the Tianjin data, which may explain this lower estimate. The difference in these estimates could also be the result of differences in containment strategies. When allowing for negative serial intervals in the Tianjin data, the mean generation time decreased to 2.90 days (95% CrI: 1.85–4.12). The sensitivity analyses showed that the assumptions made about the incubation period have only moderate impact on the results. On the other hand, assumptions made about the underlying transmission network (e.g. acknowledging possibly negative serial intervals) had a large impact on our results.

As expected, the proportion of pre-symptomatic transmission increased from 48% (95% CrI: 32–67) in the baseline scenario to 66% (95% CrI: 45–84) when allowing for negative serial intervals, for the Singapore data, and from 62% (95% CrI: 50–76) to 77% (95% CrI: 65–87) for the Tianjin data. When the incubation period is larger, it is expected that these proportions will be higher and when it is smaller, they are expected to be lower. Hence, a large proportion of transmission appears to occur before symptom onset, which is an important point to consider when planning intervention strategies. It is worth noting that the outbreak data we used were collected in the presence of intervention measures such as case isolation and quarantining of identified contacts. This means that our estimates do not necessarily reflect the natural epidemiology of COVID-19, but instead reflect what is observed in the presence of these intervention measures. It is expected that these measures reduce the proportion of symptomatic transmission, which implies that a high proportion of infections is likely to have occurred before symptom onset because isolation prevents symptomatic transmission.

We also estimated *R* for the sole purpose of illustrating the bias that occurs when using the serial interval as a proxy for the generation interval [8]. Whereas the impact was limited for our analyses, estimates based on the generation interval are larger and should be preferred to inform intervention policies. Indeed, as expected, the reproduction number was underestimated when using the serial interval distribution which is more variable than the generation interval distribution.

Tindale et al. [15] recently estimated the mean serial interval for COVID-19 to be 4.56 days (95% CI: 2.69–6.42) for Singapore and 4.22 days (95% CI: 3.43–5.01) days for Tianjin. Although these estimates are different from the ones we report, they fall within the uncertainty ranges we obtained. An important advantage of our method is that we are able to infer the generation interval distribution while allowing serial intervals to be negative. Our estimates of *R* are smaller than the ones reported by Tindale et al. [15] because we use a different estimate of the growth rate *r*. To expand, we used 0.04 for Singapore and 0.12 for Tianjin, as obtained from the initial exponential growth phase in each dataset, compared with the 0.15 used by Tindale et al. [15]. Our estimates of the serial interval are also in line with those of Du et al. [16], which estimated a mean of 3.96 days (95% CI: 3.53–4.39) and a SD of 4.75 days (95% CI: 4.46–5.07).

Another advantage of our method is that we can derive a proper variance estimate for the generation interval, in contrast to using a too large variance estimate that is obtained when using the serial interval as a proxy for the generation interval. Furthermore, from a biological point of view, we do not need to condition on the order of symptom onset times. However, when the data do not provide sufficient information on directionality of transmission, this lack of auxiliary information may cause problems for estimation.

Our study does have some limitations. First, we rely on previous estimates for the incubation period. However, our sensitivity analyses showed that changing the incubation period distribution does not have a big impact on our estimates of the generation interval distribution. Second, we do not account for incomplete or possible changes in reporting over the course of the epidemic. Incomplete reporting means that cases are missing, with this leading to incomplete transmission networks. As the underlying transmission network has a large impact on our estimates, incomplete reporting may bias our estimates. Third, we do not acknowledge changes in contact patterns and thus behavioural change, which could shape realised generation interval distributions as well as serial interval distributions (data not shown). Fourth, we do not account for contraction of the generation interval because of depletion of susceptibles. Future work should take these shortcomings into account.

In the beginning of the pandemic, infection control for the COVID-19 epidemic relied on case-based measures such as finding cases and tracing contacts. A variable that determines how effective these case-based measures are is the proportion of pre-symptomatic transmission. Our estimates of this proportion are high, ranging from 48% to 77%. This implies that the effectiveness of case finding and contact tracing in preventing COVID-19 infections will be considerably smaller compared with the effectiveness in preventing severe acute respiratory syndrome coronavirus (SARS-CoV) or Middle East respiratory syndrome coronavirus (MERS-CoV) infections, where pre-symptomatic transmission did not play an important role (see e.g [17]). As has been shown by other studies, e.g Hellwell et al. [18], it is unlikely that these measures alone will suffice to control the COVID-19 epidemic. Additional measures, such as physical distancing, are required and are already implemented in most countries.
